# Ultrasound evaluation of anterior transvaginal mesh for pelvic organ prolapse: correlation to 5-year clinical outcomes

**DOI:** 10.1007/s00192-021-04889-6

**Published:** 2021-06-29

**Authors:** Georgios Poutakidis, Anna Marsk, Daniel Altman, Christian Falconer, Edward Morcos

**Affiliations:** 1 Department of Clinical Sciences, Division of Obstetrics and Gynecology, Karolinska Institutet Danderyd Hospital, SE-176 77 Stockholm, Sweden; 2 Department of Gynecological Ultrasound, UltraGyn, Stockholm, Sweden; 3 Department of Women’s and Children’s Health, Uppsala University, Uppsala, Sweden; 4 Department of Gynecology & Obstetrics, Karolinska Institutet, Danderyd University Hospital, SE-182 88 Danderyd, Stockholm, Sweden

**Keywords:** Anterior transvaginal mesh, Ultrasound, Anatomical outcomes, Patient-reported prolapse outcomes

## Abstract

**Introduction and hypothesis:**

Vaginal prolapse mesh may effectively restore vaginal anatomy. The aim of this study was to investigate how the in vivo mesh position correlates to clinical outcomes.

**Methods:**

Seventy-one women operated on using Uphold mesh for apical pelvic organ prolapse (POP-Q, C ≥ stage II) were examined 5 years after surgery by introital-perineal 2D ultrasound in a midsagittal plane at rest and Valsalva. The horizontal line and pubis symphysis were considered the reference for all measures. Ultrasound measures were statistically compared to clinical outcomes: POP-Q, Pelvic Floor Distress Inventory (PFDI-20) and subscales [Pelvic Organ Distress Inventory (PODI-6), and Urinary Distress Inventory (UDI-6)] and the VAS scale for pain.

**Results:**

Original mesh length was preserved by 86% and correlated to improved pain as estimated by VAS scale (*r* 0.321). Valsalva was associated with a lowering of the superior and inferior mesh margins by 7.3 and 6.1 mm, respectively (*p* < 0.001) but a reduction of total mesh length by only 1 mm (30.2 ± 5.2 to 29.2 ± 4.7 mm, *p* < 0.001). Mobility of the anterior vaginal wall (bladder neck and midurethra) at Valsalva was parallel to downward movement of the mesh inferior margin (*r* 0.346 and 0.314) but inversely correlated to total UDI-6 (*r* − 0.254 and − 0.263). Mobility of the midurethra was inversely correlated to bladder emptying (PFDI-20 Question 19, *r *− 0.245).

**Conclusions:**

Five years after surgery, preserved original length of the mesh with apical support was correlated to improved anatomical and patient-reported outcomes. Mesh support to the vaginal apex was associated with improved bladder emptying and total urinary distress outcomes but not stress urinary incontinence.

## Introduction

The Uphold Lite vaginal mesh procedure for pelvic organ prolapse supports the vaginal apex and covers the upper-middle segment of the anterior vaginal wall [1, 2]. Using the Uphold kit, patient-reported outcomes and anatomical outcomes have been shown to remain stable over a 5-year period after surgery [3–5] but it is unknown how the mesh position correlates to anatomical and patient-reported outcomes. It may be difficult to clinically determine the position of the mesh postoperatively, which may be of importance if additional surgical treatments are necessary. Furthermore, there are claims of vaginal mesh contracture although reports of short-time follow-up indicated no contracture [6–8]. The value of radiologic imaging of pelvic mesh by computerized tomography CT scan and magnetic resonance imaging (MRI) is limited, and it does not provide optimal visualization of synthetic mesh implants [9–12]. Thus, ultrasound has been used in diagnosis of pelvic organ prolapse, voiding dysfunction, and evaluation of synthetic vaginal meshes for pelvic organ prolapse and urinary incontinence [9–16]. The aim of the study was to investigate whether the in vivo position of the Uphold mesh as determined by ultrasound was associated with the clinical outcomes 5 years after surgery.

## Materials and methods

All patients had been operated on for apical prolapse (POP-Q stage ≥ 2) using the Uphold™ Lite Vaginal Support System as previously described [2, 3, 17]. The Uphold™ Lite is a monofilament, microporous, and uncoated polypropylene mesh. Patients were operated on between 2012 and 2014 by two surgeons using a standardized surgical procedure [2, 3, 17]. The study was approved by the Stockholm Regional Board of Ethics at Karolinska Institutet, Stockholm, Sweden.

The pelvic organ prolapse quantification system (POP-Q) was used for anatomical assessment of prolapse: POP-Q C for the apical vaginal segment, POP-Q Ba for the anterior wall, and POP-Q TVL for assessment of the total vaginal length [18]. All patients included had suffered symptomatic apical prolapse (POP-Q stage ≥ 2). POP-Q stage 0 or 1 in the apical vaginal segment was considered an optimal anatomical outcome after surgery and was the primary outcome measure for the analysis. Exclusion criteria were current or previously treated pelvic organ cancer, cervical elongation, severe rheumatic disease, insulin-treated diabetes mellitus, connective tissue disorders, current systemic steroid treatment, and urinary incontinence.

To assess the patient self-evaluated disease-specific pelvic floor outcomes, the Pelvic Floor Distress Inventory 20 (PFDI-20) questionnaire and three subscales—Pelvic Organ Prolapse Distress Inventory-6 (POPDI-6), Urinary Distress Inventory-6 (UDI-6), and Colorectal-Anal Distress Inventory-8 (CRADI-8)—were used [19]. The total score of each subscale ranges from 0 to 100 points, and the total PFDI-20 is the sum of the three scales and ranges from 0 to 300 points. Each individual question of the POPDI-6 and UDI-6 ranges from 0 to 16.6 points out of a maximum score of 100 points per scale. PFDI-20 Question 3 was used to assess the degree of sense of vaginal bulge, Question 16 for urge urinary incontinence (UUI), Question 17 for stress urinary incontinence, and Question 19 for urinary bladder emptying. The VAS scale was used to assess the degree of pain [20]. The VAS scale is a horizontal 11-point scale where 0 indicates no pain and 10 points severe pain. The mean time of follow-up at ultrasound examination and clinical outcome assessments (POP-Q, PFDI-20, and VAS scale) was 5 years after surgery.

Introital-perineal 2D ultrasound using the Voluson™ E10-GE BT 16 level extension 1D Ultrasound System and the Voluson abdominal probe (Wide Band Convex Ultra-light Volume probe with bandwidth 2–8 MHz and a FOV of 90° V 90° × 85°) was used for all examinations. Seventy-one patients were examined by a senior consultant gynecologist at an ultrasound referral unit. Patients were examined in the supine position at rest and Valsalva (physical strain) using a standard protocol prior to initiation of the study. Sixty-six out of 71 patients were also available for repeated ultrasound examination by a second gynecologist using the same protocol to investigate the reproducibility of ultrasound measurements. All examinations were performed at the Department of Obstetrics and Gynecology, Danderyd Hospital, Stockholm, Sweden.

**Fig. 1:**
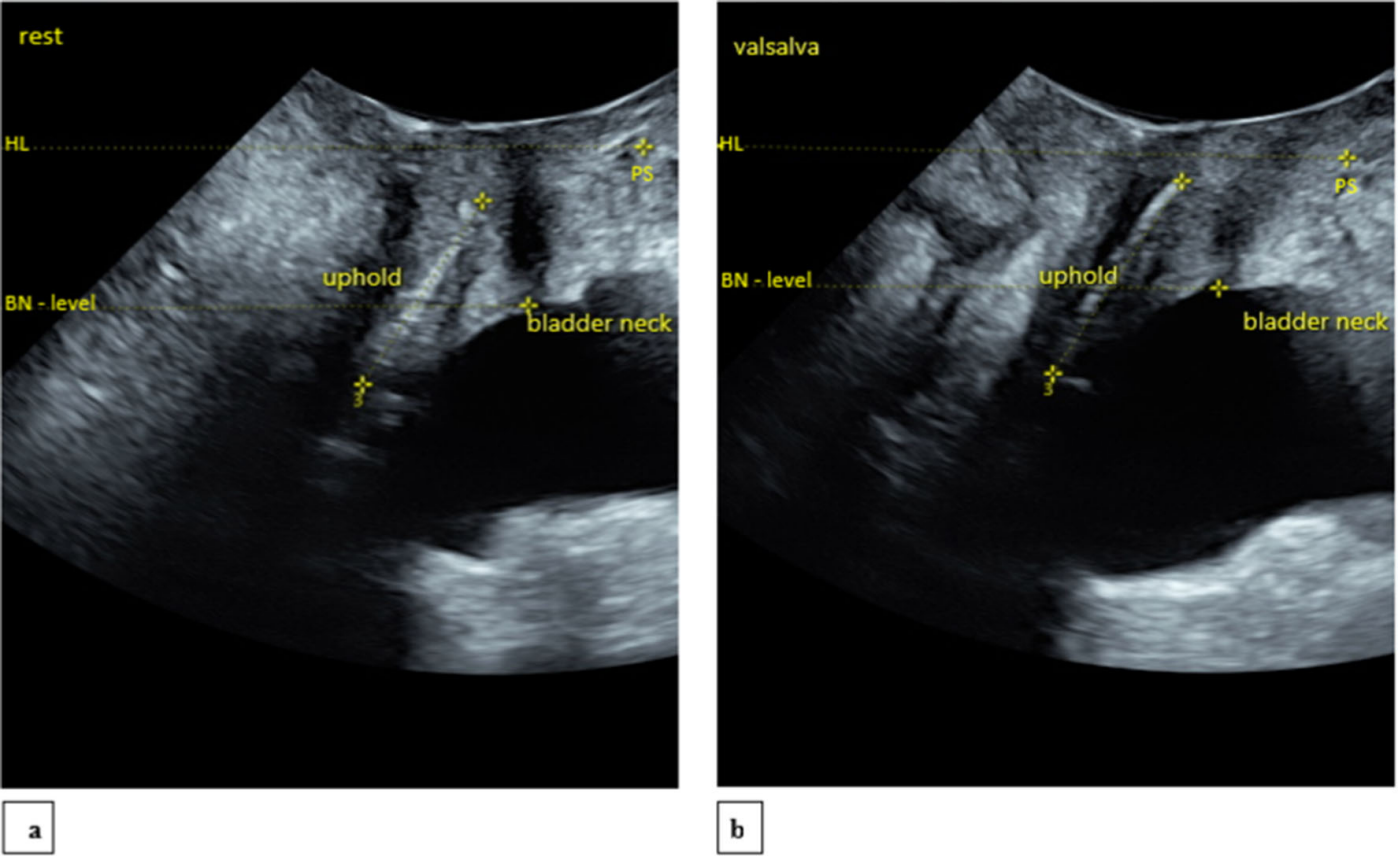
Ultrasound examination of a patient at rest (Fig. 1a) and Valsalva (Fig. 1b). HL = horizontal line, PS = pubis symphysis, BN level = bladder neck level. The total midsagittal Uphold mesh length is indicated between the upper and lower markings

At every examination, the abdominal ultrasound probe was first positioned in an introital-perineal position to capture all relevant points of measure in a single midsagittal view image [10, 12]. The pubic symphysis (PS) was subsequently identified and put to the far right of the view. When the implanted mesh (hyperdense white view), urethra, and bladder neck were identified in the same imaging field, the image was saved and measurements performed at rest and Valsalva (Fig. 1). Thus, the inferior margin of the PS was considered the fixed point, and all measures were done in only one ultrasound field. The horizontal line (HL) crossing the lower edge of the PS was considered the reference level for all ultrasound measures (10). The urinary bladder neck, also known as the urethro-vesical junction, is indicated as BN and identified as the point of meeting between the most inferior part of the urinary bladder and the most superior part of the urethra. The BN level was drawn horizontally across the BN and is parallel to the HL; it was considered to be basic in all measurements. All measurements of the mesh, bladder neck, and urethra were estimated in millimeters (mm) from a midsagittal midline position in the ultrasound image view [10, 12].

**Fig. 2:**
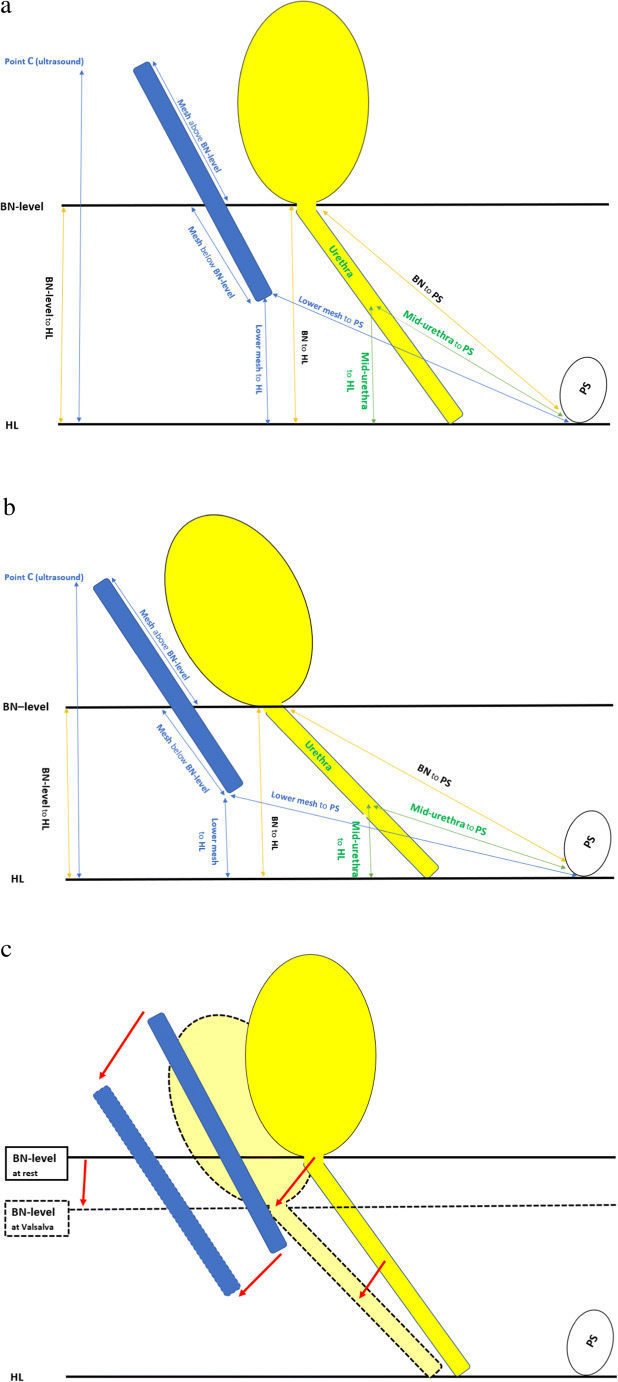
(a) All ultrasound measures at rest; (b) all measures at Valsalva. (c) All measures at rest (whole line) and Valsalva (hatched line). All figures are constructed after all measures shown in Table 2. HL: horizontal line. PS: pubis symphysis. BN: bladder neck. BN level: bladder neck level

All ultrasound outcomes at rest and Valsalva are shown in Table 2 and illustrated in Fig. 2. Total mesh midsagittal length, mesh length above and below the BN level, and distance from the mesh inferior margin to the PS and HL were estimated. The point C ultrasound was considered as the distance from the superior margin (most upper edge) of the mesh to the HL. The distance from the BN to the PS and HL was also estimated. Furthermore, total urethra length and distance from a mid-urethra point to the PS and HL were estimated. Distances measured above the HL were considered negative values whereas distances below the HL as positive values.

### Statistical analyses

Paired sample *t*-test was used to test the means of two metric variables of the same ultrasound measures at rest and Valsalva. Repeated measures ANOVAs were used to test total values at rest vs. Valsalva and to compare the measures between observers. Repeated measures ANOVA was also used to compare changes in measurements between rest and Valsalva and between observers. The two-way intra-class correlation coefficient (ICC) was used to determine the absolute agreement (single measures) between observers. The ICC was estimated at each single ultrasound measurement at rest and Valsalva. ICC values < 0.40 were considered poor agreement, 0.40–0.59 fair agreement, and 0.60–0.74 good inter-observer agreement. Bivariate correlations were statistically tested by Pearson’s correlation coefficient and Pearson’s *r* ranges between +1 and − 1, where 1 is total positive linear correlation, 0 is no linear correlation, and − 1 is total negative linear correlation according to the Cauchy-Schwarz inequality. Pearson’s *r* values of −0.20 − −0.39 to + 0.20 – 0.39 and at least 47 observations with a power of analysis of 80% were considered significantly correlated [21]. The Bland-Altman plot was used to scatterplot the differences in ultrasound measures between two observers. All statistical analyses were performed using predictive analysis software (IBM^@^SPSS© Statistics, Version 25, Inc., Chicago, IL, USA, 2017). All missing data were considered missing without imputation of data.

## Results

### Demographic and surgical characteristics

Preoperative patients’ medical and surgery characteristics are described in detail in Table 1. The mean age was 65.7 ± 9.7 years, and the mean BMI was 26.5 ± 3.4 kg/m^2^. Parity was at a mean of 2.4 ± 1.3, and vaginal deliveries were 96% of all deliveries. Sixty-seven out 71 (94.4 ± 2.7%) patients were in menopause, and 33 out of 71 (46.5%) patients used at least one HRT preparation. A total of 16 patients out of 60 (26.7 ± 5.7%) reported no disease, whereas 30 patients (50.0 ± 6.5%) reported at least 1 cardiovascular disease and 14 out of 60 patients (23.3 ± 5.5%) reported other chronic diseases. Thirteen patients (18.3 ± 4.6%) had previous hysterectomy, and 42 patients (59.2 ± 5.8%) had received at least one previous prolapse surgery prior to the primary surgery with the Uphold™ Lite mesh. All patients (*n* = 71) received the Uphold™ Lite mesh, and 12 out of 71 patients (16.9 ± 4.5%) were also operated on by anterior colporraphy at the same time the mesh was implemented.

**Table 1:** Preoperative demographics and surgery characteristics

	***Mean ± SD***	***Confidence interval*** *(CI)*	***n***
**Age (years)**	65.7 ± 9.7	63.4–68.0	71
**Height (cm)**	165.3 ± 6.0	163.9–166.8	71
**Weight (kg)**	72.6 ± 10.8	70.0–75.1	71
**Body mass index (kg/m**^**2**^**)**	26.5 ± 3.4	25.7–27.3	71
**Parity**	2.4 ± 1.3	2.1–2.6	71
Vaginal delivery	2.3 ± 1.3	2.0–2.6	
Instrumental delivery	0.2 ± 0.4	0.1–0.3	
Cesarean section delivery	0.1 ± 0.3	0.0–0.1	
	***Number (%)***	***Confidence interval*** *(CI)*	***n***
**Menopause**			
Menopause	94.4 ± 2.7%	87.2–98.1%	67
Still having menstruation	5.6 ± 2.7%	1.9–12.8%	4
**Hormonal replacement therapy (HRT)**			
None	53.5 ± 5.9%	42–64.8%	38
Hormonal intrauterine device	0	0	0
Estrogen (tablet, patch)	7 ± 3.0%	2.7–14.7%	5
Local estrogen (vaginal)	31 ± 5.5%	21.2–42.3%	22
Combined HRT	8.5 ± 3.3%	3.6–16.6%	6
**Somatic diseases**			
No diseases	26.7 ± 5.7%	16.8–38.8%	16
Cardiovascular diseases	50.0 ± 6.5%	37.6–62.4%	30
Other diseases	23.3 ± 5.5%	14.0–35.1%	14
**Previous pelvic floor surgeries**			
Previous hysterectomy	18.3 ± 4.6%	10.7–28.5%	13
Still having uterus	81.7 ± 4.6%	71.5–89.3%	58
Previous prolapse surgery (recurrence)	59.2 ± 5.8%	47.5–70.0%	42
No previous prolapse surgery	40.8 ± 5.8%	30.0–52.2%	29
**Operation**			
Uphold	83.1 ± 4.5%	73.1–90.4%	59
Uphold + anterior colporraphy	16.9 ± 4.5%	9.6–26.9%	12

### Ultrasound measures at rest and Valsalva

Outcomes of all ultrasound measurements at rest and Valsalva using paired sample *t*-test are shown in Table 2 and illustrated in Fig. 2. Total midsagittal length of the mesh was estimated at 30.2 ± 5.2 mm at rest and 29.2 ± 4.7 mm at Valsalva. Results indicate a decrease of total mesh length by 1 mm (3.3%) at Valsalva compared to rest (*p* = 0.04). There was a significant shortening of the mesh length above the BN level between rest and Valsalva by 2.8 mm (*p* = 0.017). However, there was no significant change of mesh length below the BN level at Valsalva (13.8 ± 9.2 to 15 ± 8.5 mm, *p* = 0.246). The point C ultrasound was significantly lower, i.e., moved downward at Valsalva by + 7.3 mm [(−42.8 ± 11.5 to −35.5 ± 11.2 mm), *p* < 0.001].

**Table 2:** All measures from 2D ultrasound at rest and Valsalva in mm (*n* = 71)

	**2D measures at rest**		**2D measures at Valsalva**		
	***Mean ± SD (CI)***	*n*	***Mean ± SD (CI)***	*n*	***p*** **value**
**Total mesh length**	30.2 ± 5.2 (28.99–31.43)	71	29.2 ± 4.7 (28.06–30.32)	70	0.040
**Mesh length above BN level**	16.6 ± 10.5 (14.16–19.11)	71	13.8 ± 9.7 (11.46–16.06)	71	0.017
**Mesh length below BN level**	13.8 ± 9.2 (11.59–15.93)	71	15 ± 8.5 (13.04–17.04)	71	0.246
**Mesh lower edge to PS**	41.1 ± 10.1 (38.73–43.53)	71	43.2 ± 12.5 (40.22–46.15)	71	0.093
**Mesh lower edge to HL**	−15.7 ± 9.7 (−18.53 – −12.84)	47	−9.6 ± 8.6 (−12.12 – −7.07)	47	0.001
**Point C ultrasound (mesh upper edge to HL)**	−42.8 ± 11.5 (−45.54 – −40.12)	71	−35.5 ± 11.2 (−38.17 – −32.85)	71	0.001
**BN to PS**	35.9 ± 5.34 (34.68–37.21)	71	35.3 ± 6.3 (−36.84 – −33.84)	71	0.303
**BN level to HL**	−26.2 ± 4.9 (−27.36 – −25.03)	71	−20.4 ± 5.6 (−21.69 – −19.04)	71	0.001
**Total urethra length**	31.4 ± 3.9 (30.47–32.29)	71	29.8 ± 3.8 (28.91–30.69)	71	0.001
**Mid-urethra to PS**	23.1 ± 4.9 (21.92–24.25)	71	21.7 ± 5.6 (20.43–23.06)	71	0.064
**Mid-urethra to HL**	−10.9 ± 3.6 (−11.73 – −10.04)	71	−7.6 ± 3.5 (−8.41 – −6.73)	70	0.001

Paired sample *t*-test was used, *p* < 0.05 was considered significant

HL = horizontal line, PS = pubis symphysis, BN = bladder neck, BN level = bladder neck level

The distance between the inferior margin of the mesh and the PS was not significantly changed between rest and Valsalva (*p* = 0.093). However, the distance between the inferior margin of the mesh and the HL was shortened at Valsalva (−15.7 ± 9.7 to −9.6 ± 8.6 mm; *p* < 0.001). This shortening of distance between the inferior margin of the mesh and the HL was paralleled by a shortening of the distance between the BN level and the HL (−26.2 ± 4.9 to −20.4 ± 5.6 mm; *p* < 0.001), i.e., simultaneous downward movement of the bladder neck and mesh inferior margin.

The BN level was significantly lowered at Valsalva, i.e., proximal to the HL by 5.8 mm (−26.2 ± 4.9 to −20.4 ± 5.6 mm, *p* < 0.001). The total urethral length and the distance between the point of the mid-urethra to the HL were also shortened at Valsalva [(31.4 ± 3.9 to 29.8 ± 3.8 mm) and (−10.9 ± 3.6 to −7.6 ± 3.5 mm), respectively; *p* < 0.001]. Neither the distance from the BN nor the mid-urethra to PS was significantly changed at Valsalva (*p* = 0.303 and 0.064, respectively).

Results of statistical analysis using repeated measures ANOVA for comparison of ultrasound measures at rest and Valsalva and between two observers are shown in Table 3. Comparing between observers when detecting changes between rest and Valsalva (i.e., the difference between measured values at rest and Valsalva) showed no significant change (*p* = 0.33–0.983). The ICC absolute agreement between observers (single measures) ranged from 0.54–0.69 (fair–good degree) for all measurements concerning the mesh at rest. The ICC was also fair–good (0.41–0.66) for all mesh-related measurements at Valsalva except the distance between the inferior margin of the mesh and the PS (ICC 0.13).

**Table 3:** Repeated ultrasound measurements by two different observers at rest and Valsalva (*n* = 66)

	**Observer 1**		**Observer 2**			
	**Rest** *mean ± SD*	**Valsalva** *mean ± SD*	**Rest** *mean ± SD*	**Valsalva** *mean ± SD*	***P*** **value** **(total measures)**	***P*** **value** **(between measures)**
**Total mesh length**	30.20 ± 5.14	29.20 ± 4.8	29.7 ± 4.34	28.7 ± 4.62	0.005	0.983
**Mesh length above BN level**	16.05 ± 10.4	13.7 ± 9.73	15.7 ± 8.21	14.2 ± 8.9	0.005	0.357
**Mesh length below BN level**	14.0 ± 0.9.32	15.2 ± 8.56	14.3 ± 8.13	14.3 ± 8.34	0.352	0.386
**Mesh lower edge to PS**	41.0 ± 10.13	42.7 ± 12.41	35.3 ± 10.12	35.4 ± 17.64	0.486	0.494
**Mesh lower edge to HL**	−15.2 ± 9.1	−9.6 ± 8.71	−15.5 ± 8.01	−7.2 ± 8.2	0.001	0.333
**Point C ultrasound (upper mesh to HL)**	−42.8 ± 11.3	−34.1 ± 11.20	−42.4 ± 9.3	−34.5 ± 10.1	0.001	0.618
**BN to PS**	36.1 ± 5.29	35.3 ± 6.15	32.5 ± 5.53	31.0 ± 6.67	0.019	0.487
**BN level to HL**	−26.2 ± 4.94	−20.7 ± 5.44	−26.7 ± 5.14	−20.3 ± 6.57	0.001	0.358
**Total urethra length**	31.4 ± 3.89	29.7 ± 3.82	31.9 ± 4.8	30.3 ± 5.29	0.001	0.921
**Mid-urethra to PS**	23.3 ± 4.94	21.6 ± 5.58	20.3 ± 5.92	19.3 ± 6.71	0.008	0.451
**Mid-urethra to HL**	−10.9 ± 3.59	−7.6 ± 3.63	−12.7 ± 3.83	−8.9 ± 4.38	0.001	0.399

Repeated measures ANOVA; *p* < 0.05 was considered significant. *P*-values (total measures) indicate statistically significant difference for each single measure at rest and Valsalva and between observers, whereas *p*-values (between measures) indicate statistical significance or not for the differences in measures between rest and Valsalva for each single measure and between observers

### Ultrasound measures and clinical outcomes

Correlations between the ultrasound measures at Valsalva and clinical outcomes are described in Table 4. In the present study, POP-Q anatomical outcomes were 97% for the apical (POP-Q point C stage 0–1) and 93% for the anterior wall (POP-Q point Ba stage 0–1), respectively. At Valsalva, there was no correlation between ultrasound measures (point C ultrasound, BN level, and midurethra) and POP-Q outcomes or the sense of symptomatic pelvic organ prolapse, i.e., vaginal bulge as estimated by PFDI-20 Question 3 (Table 4). Also no correlation was found at rest.

**Table 4:** Correlation of ultrasound measures at Valsalva and clinical outcomes

		Ultrasound Point C - Ultrasound		BN−level (bladder neck level)		Midurethra	
Anatomical outcomes POP-Q	*mean ± SD*	Pearson's *r*	*n*	Pearson's *r*	*n*	Pearson's *r*	*n*
Apical, C	−5.4±1.37	−0.149	68	−0.025	68	−0.109	67
*Anterior wall*, Ba	−2.6±1.28	−0.039	68	−0.103	68	−0.130	67
*Vaginal length*, TVL	8.8±1.59	0.184	68	0.074	68	0.141	67
Patient-reported outcomes PFDI-20							
POPDI-6 (Total)	16.0±15.88 / (100)	−0.093	67	−0.153	67	−0.235	66
*POPDI-6 Question 3* (*sense of bulge)*	0.7±4.54 / (16.7)	0.094	67	0.008	67	−0.067	66
UDI-6 (Total)	13.7±15.58 / (100)	−0.234	67	−0.254*	67	−0.263*	66
*UDI-6, Question 16* (Urge incontinence)	1.0±1.30 / (16.7)	−0.124	68	−0.220	68	−0.167	67
*UDI-6, Question 17* (stress incontinence)	1.2±1.20 / (16.7)	−0.262*	69	−0.197	69	−0.205	68
*UDI-6, Question 19* (bladder emptying)	0.7±1.07 / (16.7)	−0.226	68	−0.185	68	−0.245*	67
PFDI-20 (Total)	50.4±42.18 / (300)	−0.211	62	−0.169	62	−0.244	61
Pain (VAS-scale, 0−10)	0.5±1.25 (10)	−0.245*	67	−0.250*	68	−0.307*	68

Analysis of bivariate correlation by Pearson correlation coefficient. *Significant Pearson’s *r* correlation

Only patients with available ultrasound measures and clinical outcomes were included

POP-Q measures in cm, UDI-6 (100 points), POPDI-6 (100 points), and PFDI-20 total (300 points), whereas individual PFDI-20 questions (16.66 points for each question). VAS scale (0–10) was used for evaluation of pain

In a separate analysis, we investigated the mesh compliance to downward movement of the anterior vaginal wall at Valsalva. Two levels were considered for analysis of the anterior vaginal wall downward movement, the BN level and the mid-urethra HL, to be statistically tested for the mesh levels. Downward movements of the BN level and mid-urethra were parallel to downward movement of the mesh inferior margin (Pearson’s *r* 0.346 and *r* 0.314, respectively), but there was no correlation to point C ultrasound, i.e., mesh superior margin (*r* 0.059 and *r*0.088, respectively) or total mesh length (*r* 0.187 and *r* 0.042, respectively).

The BN level and mid-urethra downward movement at Valsalva were inversely correlated to the total UDI-6 (*r* − 0.254 and *r* − 0.263, respectively), i.e., the lower the BN level and mid-urethra were, the higher the UDI-6 score (Table 4). There was also a negative correlation between the point C ultrasound and the inferior margin of the mesh to PFDI-20 Question 17 score, i.e., SUI (*r* − 0.262 and *r* − 0.304, respectively). Also, the mid-urethra was negatively correlated to bladder emptying as estimated by PFDI-20 Question 19 (*r* − 0.245). In a sub-analysis excluding all patients having previously received a mid-urethral sling, 25 patients had stress urinary incontinence whereas 30 did not (PFDI-20, Question 19). Using independent sample *t*-test, the BN level was significantly correlated to stress urinary incontinence (*p* = 0.038).

We also investigated the correlation of ultrasound measures and estimated pain by VAS scale. The VAS pain score was estimated as 0.5 ± 1.25, and 59 out of 68 patients (87%) had a VAS score 0–1 points. The total mesh length was inversely correlated to pain (Pearson’s *r* − 0.321) indicating that preserved mesh length may be correlated to less pain. Furthermore, the apical and anterior vaginal wall anatomical points estimated by ultrasound (point C ultrasound, BN level, and midurethra) were significantly correlated to the low VAS scale score (Pearson’s *r* − 0.245, *r* − 0.250 and *r* − 0.307, respectively) (Table 4). This may indicate that the higher the apical and the anterior wall anatomical ultrasound points are, the lower the VAS pain score.

## Discussion

In this 5-year follow-up study, we evaluated the Uphold anterior vaginal mesh by 2D ultrasound image to investigate the mesh correlation to anatomical and patient-reported clinical outcomes. We found no mesh contracture, and the mesh still preserved 86% of the original mesh length and a dynamic compliance to movement of the apical and anterior vaginal segments. Anatomical mesh support of the apical and anterior vaginal wall may indicate a positive correlation to improved patient-reported urinary distress symptoms as estimated by the total UDI-6. Our results suggest that the higher the bladder neck is, the better the emptying of the urinary bladder and that low bladder neck mobility may correlate to stress urinary incontinence. However, mesh support to the urinary bladder neck had no correlation to the outcome of stress urinary incontinence.

Five years after surgery, we found total preserved mesh midsagittal length to be 86% of the original pre-implantation length. These results correlate to previous studies showing a retained length of 86% 1 month and 89% 1 year after surgery [8] and suggest that when used for trocar-guided apical repair, shortening of the mesh in the midsagittal plane is limited. Furthermore, preserved mesh length correlated to less pain estimated by the VAS score.

We also investigated the mesh dynamic mobility in relation to the apical and anterior vaginal wall segments. Although a downward movement of mesh superior and inferior margins were 7.3 and 6.1 mm at Valsalva, the total mesh length was only reduced by 1 mm. The downward movement of the anterior vaginal wall (bladder neck and midurethra) was significantly correlated to movement of the mesh part below the bladder neck but did not correlate to the upper part of the mesh. These results suggest that a dorsal and inferior movement of the mesh with a minimal reduction of total midsagittal length and limited downward movement of the apical part of the mesh may still support the apical vaginal segment although the mesh was in dynamic compliance with the movement of the anterior vaginal compartment.

Imaging of the upper part of the mesh at Valsalva may be useful to understand the anchoring level of the mesh in relation to the sacrospinous ligament. During surgery, the mesh is passed through the sacrospinous ligaments and adjusted to support the uterus or vaginal vault to the sacrospinous ligament level. However, there is no way to standardize the amount of tension placed during adjustment of the mesh, which is based on surgeon experience. There is a lack of knowledge on how to prevent anterior wall prolapse recurrence and avoid over-correction in pelvic reconstructive surgery. However, determining the relation of a mesh to the bladder neck and distance between the mesh lower margin and the pubis symphysis level may be useful when clinically assessing recurrence of anterior pelvic organ prolapse [22]. Optimal anatomical outcomes were found in 97% for the apical and 93% for the anterior wall segments (POP-Q Ba stage 0–1). The lack of correlation between the ultrasound mesh position and POP-Q apical and anterior vaginal wall outcomes may be explained by the high optimal anatomical outcomes as evaluated by the POP-Q (97% and 93% for the apical and anterior wall, respectively). These results may also explain why no correlation was found between ultrasound measures and patient-reported sense of vaginal bulge as estimated by POPDI-6 Question 3.

Bladder neck and urethral mobility as well as positioning of midurethral slings have been studied to investigate stress urinary incontinence and outcomes after incontinence surgery [14, 15]. We found that the mobility of the bladder neck and urethral mobility were inversely correlated to the total urinary distress symptoms, bladder emptying, and stress urinary incontinence in patients having had apical mesh surgery. There was no correlation between mobility of the bladder neck and urethra to the total pelvic organ prolapse or pelvic floor distress inventories. However, inverse correlation was found between mesh position and stress urinary incontinence.

Strengths of our study include a standardized surgical technique using an identical mesh kit and only two surgeons that performed all surgeries. Ultrasound examinations were done using an identical examination protocol. We recognize that a control group of women having undergone non-mesh augmented pelvic organ prolapse repair and/or a group with recurrent prolapse would have been a valuable addition to our understanding of how mesh and adjacent lower urinary tract anatomical positions may correlate to outcomes. Although the product has been withdrawn from the market, there is still a need for clinical evaluations to determine long-term effects of this and other mesh-based products used for pelvic reconstructive surgery [23]. However, our results may be useful for optimizing prolapse surgery and can be a valuable addition to the clinical examination in the assessment of patients with pelvic floor dysfunction who have previously had mesh surgery.

## Conclusion

The 5-year follow-up results which combined ultrasound and clinical outcomes indicate preserved mesh length with anatomical support of the apical and anterior vaginal segments, and mesh mobility compliance was correlated to disease-specific outcomes including improved pelvic organ prolapse, urinary distress, and pain outcomes at the long term. Results may be useful for optimizing prolapse surgery using the sacrospinous ligament level correction or recurrent urogynecological surgeries.
